# Supplementary material for: Patterns of Intron Gain and Loss in Fungi
Source: PLoS Biol. 2004 Nov 30;2(12):e422. doi: 10.1371/journal.pbio.0020422 (PMC532390; doi:10.1371/journal.pbio.0020422)
Supplement: Table S1 — Also available at http://genes.mit.edu/NielsenEtAl/. (4.3 MB ZIP). [file pbio.0020422.st001.zip › NielsenEtAl/html/521.html]

AN9527.1.NCU00147.1.MG06443.1.FG02600.1


```
 CLUSTAL W (1.82) Multiple Sequence Alignments - Introns Inserted


Sequence 1: NCU00147.1	178 aa
Sequence 2: MG06443.1	177 aa
Sequence 3: AN9527.1	178 aa
Sequence 4: FG02600.1	211 aa
Alignment Length: 213 aa
Number Identitical Residues: 89 aa
Alignment Score (without introns) 4537


MG06443.1 	----------------~-----------------MKAYFYDDL--P0GDQREPHDSGKAV
NCU00147.1	----------------~-----------------MKAYFYDNL--P0GDQRLPHDSGKEV
FG02600.1 	MDGTFEIQWSVCHESC2NHASLLLRWPSSPYTLILLPDLSTNKKSQ~GDQRLPHNSGKPV
AN9527.1  	----------------~-----------------MKAYWYDNK--P0GDQREPHDSGRPV
          	                                  : .    :     **** **:**: *

MG06443.1 	DTDGLASIGVLYFHLPDVSSVDSLAASRGYRNRDEITVSPEKMGDAYEDKVKSFFSEHLH
NCU00147.1	TVAELEKVGVLYFRFPDVEGVNTLAAERGYKNRDEIIVSPEKMGAIYETKVRQFFDEHLH
FG02600.1 	SVDDLMNIGVYYYHLPELEQVDNLSKEREYKNRDEITVSPQAMGDIYETKVKSFFAEHLH
AN9527.1  	SEDYLASLGVIYRHFPELSDVDALAKERGYKNRDEITVSPATMGEAYEDKVKMFFAEHLH
          	    * .:** * ::*::. *: *: .* *:***** ***  **  ** **: ** ****

MG06443.1 	EDEEIRYIRDGHGYFDVRDRADRWVRIHLDKGDLIILPAGIYHRFTTDDSN0YIQAMRLF
NCU00147.1	EDEEIRYIRDGAGYFDVRNEGDEWIRIKLVKDDLIILPAGIYHRFTTDDTN0YIQAMRLF
FG02600.1 	EDEEIRYILGGRGYFDVRSKDDDWVRVLLEKNDLLILPPGIYHRFTTDESN0FVHAMRLF
AN9527.1  	EDEEIRYIRDGEGYFDVRGKEDEWVRIRLVKDDLIILPAGIYHRFTTDNKN0YIKAMRLF
          	******** .* ******.. * *:*: * *.**:***.*********:.* :::*****

MG06443.1 	KDEPKWTPLNRVPELEDNEHRKKYVDEFLEGCSRS-
NCU00147.1	KEEPKWTPLNRTEGLDENPYRQEYVTQFLKAQAEQA
FG02600.1 	KEDPKWTPLNRGPDVDKNEHRQEYVKQFIGESTE--
AN9527.1  	QEEPKWTPLNRAPELDENQHRKSYLEGLTATSIAAN
          	:::********   ::.* :*:.*:  :
```
